# Artificial Intelligence‐Driven Automated Design of Anterior and Posterior Crowns Under Diverse Occlusal Scenarios

**DOI:** 10.1111/jerd.70029

**Published:** 2025-09-05

**Authors:** Nan Hsu Myat Mon Hlaing, Gülce Çakmak, Duygu Karasan, Sung‐Jin Kim, Irena Sailer, Jae‐Hyun Lee

**Affiliations:** ^1^ Department of Prosthodontics Seoul National University School of Dentistry Seoul Republic of Korea; ^2^ Department of Reconstructive Dentistry and Gerodontology School of Dental Medicine, University of Bern Bern Switzerland; ^3^ Department of Prosthodontics, Geriatric Dentistry and Craniomandibular Disorders Charité‐Universitätsmedizin Berlin, Corporate Member of Freie Universität Berlin and Humboldt‐Universität Zu Berlin Berlin Germany; ^4^ Division of Fixed Prosthodontics and Biomaterials, Clinique Universitaire de Médecine Dentaire (CUMD) University of Geneva Geneva Switzerland; ^5^ Dental Research Institute Seoul National University School of Dentistry Seoul Republic of Korea

**Keywords:** artificial intelligence, computer‐aided design, dental occlusion, dental prosthesis design, dental restoration

## Abstract

**Objective:**

To evaluate the impact of occlusion type and artificial intelligence‐based computer‐aided design (CAD) software on the geometric accuracy and clinical quality of auto‐generated anterior and posterior crown designs.

**Methods:**

Five typodont models representing various occlusion types (normal, Class I anterior diastema, Class II division 1, Class II division 2, and Class III anterior crossbite occlusion) underwent crown preparation for the maxillary right central incisor and first molar. Ten sets of intraoral scans were obtained from each prepared model, and crown designs were automatically generated using two software programs: deep learning‐based (DL; Dentbird) and conventional automated (CA; Auto Workflow, 3Shape) (*n* = 10). Surface deviations between the crown designs and preoperative tooth morphology were quantified using root mean square (RMS) values. Clinical crown quality was assessed using World Dental Federation (FDI) criteria. Scheirer–Ray–Hare and Fisher's exact tests were conducted (*α* = 0.05).

**Results:**

Significant differences in surface deviation and clinical quality were observed between the various occlusion and software types. The DL group demonstrated higher RMS values than the CA group (*p* < 0.001). However, DL‐generated crowns were of significantly better clinical quality (FDI scores) than CA‐generated crowns, particularly for posterior teeth, in terms of marginal adaptation, proximal contacts, and anatomical form and contour (*p* < 0.05). The DL group demonstrated generally favorable outcomes when designing crowns for normal occlusion, but outcomes were less satisfactory when designing anterior crowns with diastemas.

**Conclusions:**

Occlusal scenarios influenced the surface deviation and quality of auto‐generated anterior and posterior crown designs. DL software produced higher‐quality molar designs than CA software.

**Clinical Significance:**

Automated crown design outcomes depend on occlusal scenarios and CAD software selection. DL‐based CAD software demonstrated superior clinical quality, particularly for posterior crowns, indicating higher clinical suitability. However, further software refinement is needed to consistently produce clinically acceptable crowns under diverse occlusal conditions, such as anterior diastemas.

## 1 Introduction

Proper occlusion is pivotal in dentistry as it ensures optimal masticatory function and maintains oral health [1, 2]. Accurate diagnosis and effective management of the relationship between the upper and lower jaws are therefore essential for successful treatment planning and outcomes [3]. Full coverage crowns are commonly used to restore teeth with extensive defects, reproducing clinical crown morphology and thereby contributing to occlusal function [4, 5]. If the crown design fails to adequately replicate an acceptable occlusal morphology, discrepancies may arise, potentially reducing masticatory efficiency [6]. Therefore, accurate crown design is crucial to support aesthetics, oral functionality, and overall occlusal integrity; this emphasizes the importance of considering opposing teeth and individual occlusal patterns during crown design [7].

Despite technological advancements, reproducing tooth morphology and accommodating unique occlusal characteristics remains challenging [8]. Computer‐aided design and computer‐aided manufacturing (CAD–CAM) systems have transformed prosthetic workflows, improving design quality and efficiency, and reducing the labor burden on dental professionals [7, 8, 9, 10]. Conventional CAD methods, such as those using library tooth forms or referencing original tooth morphology, still often require operator intervention [11, 12]. To overcome these challenges, automated workflows have recently been integrated into CAD software, enhancing consistency and reducing the need for manual input [10, 13]. Artificial intelligence (AI), particularly through deep learning (DL), has significantly contributed to these advancements. In digital dentistry, DL systems using convolutional neural networks (CNNs) [14, 15] and generative adversarial networks (GANs) [7, 9, 16] have garnered attention. This DL‐based automated CAD software can design individualized crowns without operator guidance, minimizing human error and improving the accuracy of tooth morphology and occlusal alignment [7, 9, 16, 17, 18, 19]. As clinical awareness has increased, AI‐driven dental software has been widely adopted. Applications include veneers, inlays, onlays, single and partial crowns, dentures, and occlusal devices [8, 9, 10, 13, 16, 17, 18, 19, 20, 21, 22, 23, 24, 25, 26, 27, 28].

While the performance of automated CAD systems for posterior crowns has been widely investigated [9, 10, 16, 17, 18, 19, 20, 22, 23, 27, 28], anterior crown designs remain understudied [20]. Moreover, most studies have focused on ideal occlusal scenarios, overlooking clinically common conditions such as anterior crossbite, deep bite, diastema, and Class III malocclusion. In clinical practice, dentists frequently encounter patients with various malocclusions [29]; thus, it is essential to verify whether automated CAD software programs used in restorative dentistry can produce acceptable outcomes not only under ideal occlusal conditions but also in diverse malocclusion scenarios. To address these gaps, the aim of this study was to compare the surface deviations of anterior and posterior crown designs produced by two automated CAD software systems, and to examine the influence of different occlusion types on design quality and aesthetics. The first null hypothesis was that no differences would exist among the five occlusion types, as assessed using surface deviation and quality scores. The second null hypothesis was that the two automated CAD software programs would not differ significantly in their crown design performance, regardless of occlusion type.

## 2 Materials and Methods

### 2.1 Study Design and Sample Size Calculation

**FIGURE 1:**
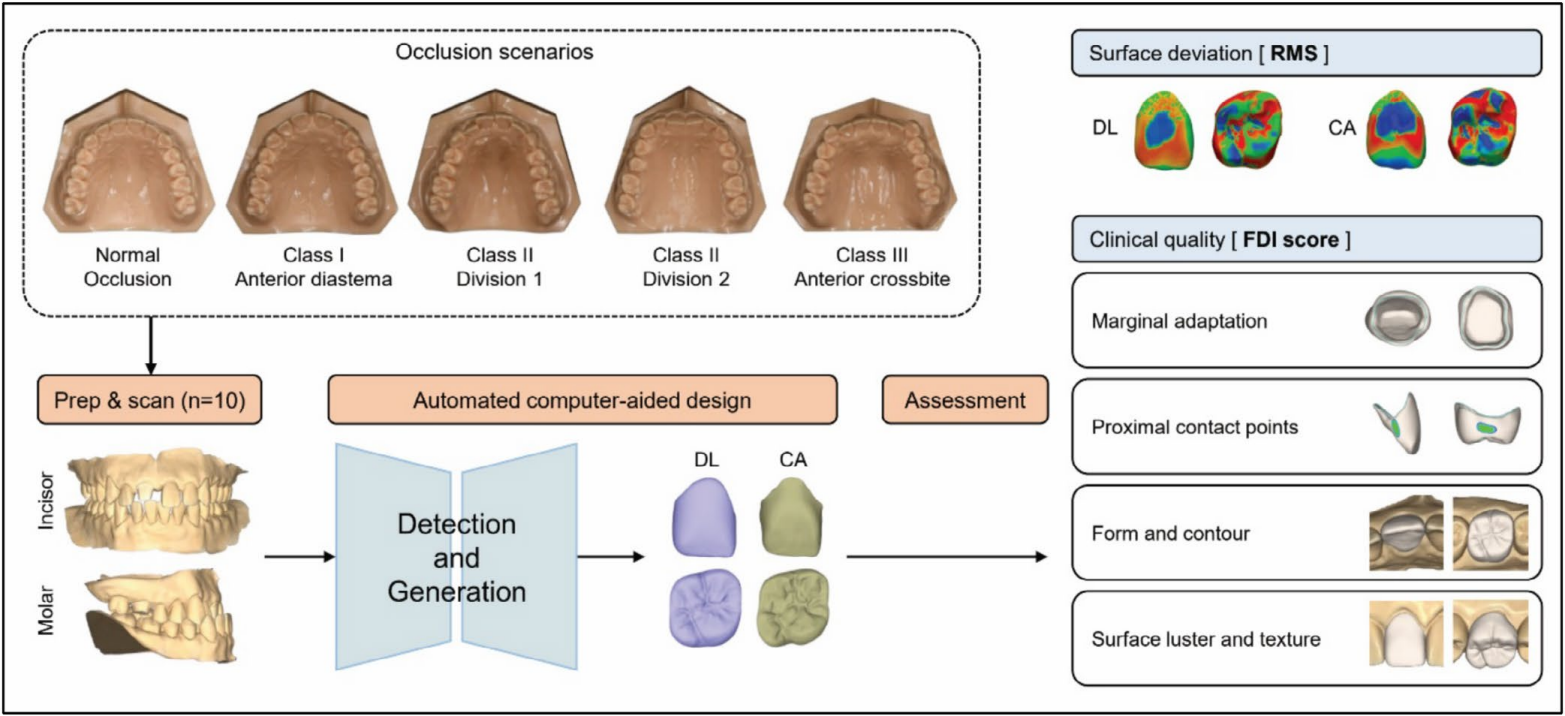
Flowchart of the study. CA, conventional automated computer‐aided design; DL, deep learning‐based computer‐aided design; FDI, World Dental Federation; RMS, root mean square.

The overall workflow of the study is shown in Figure 1. Five typodont models representing different occlusion types were used to evaluate crown designs of the maxillary right first molar and the maxillary right central incisor, generated by two automated CAD software programs. Evaluations were performed using two distinct approaches: a quantitative surface deviation analysis, comparing the designs to pre‐preparation reference forms using root mean square (RMS) values, and a qualitative evaluation based on World Dental Federation (FDI) criteria.

A pilot test (*n* = 3 per group) was initially conducted to estimate the effect size required for sample size determination. Using G*Power software (v. 3.1.9.4; GPower, Hinnerup, Denmark) [30], an effect size of 0.52 was calculated based on the pilot test data. To achieve a statistical power > 80% with an *α* = 0.05, a final sample size of 10 per group was determined.

### 2.2 Preparation of Typodont Models

**FIGURE 2:**
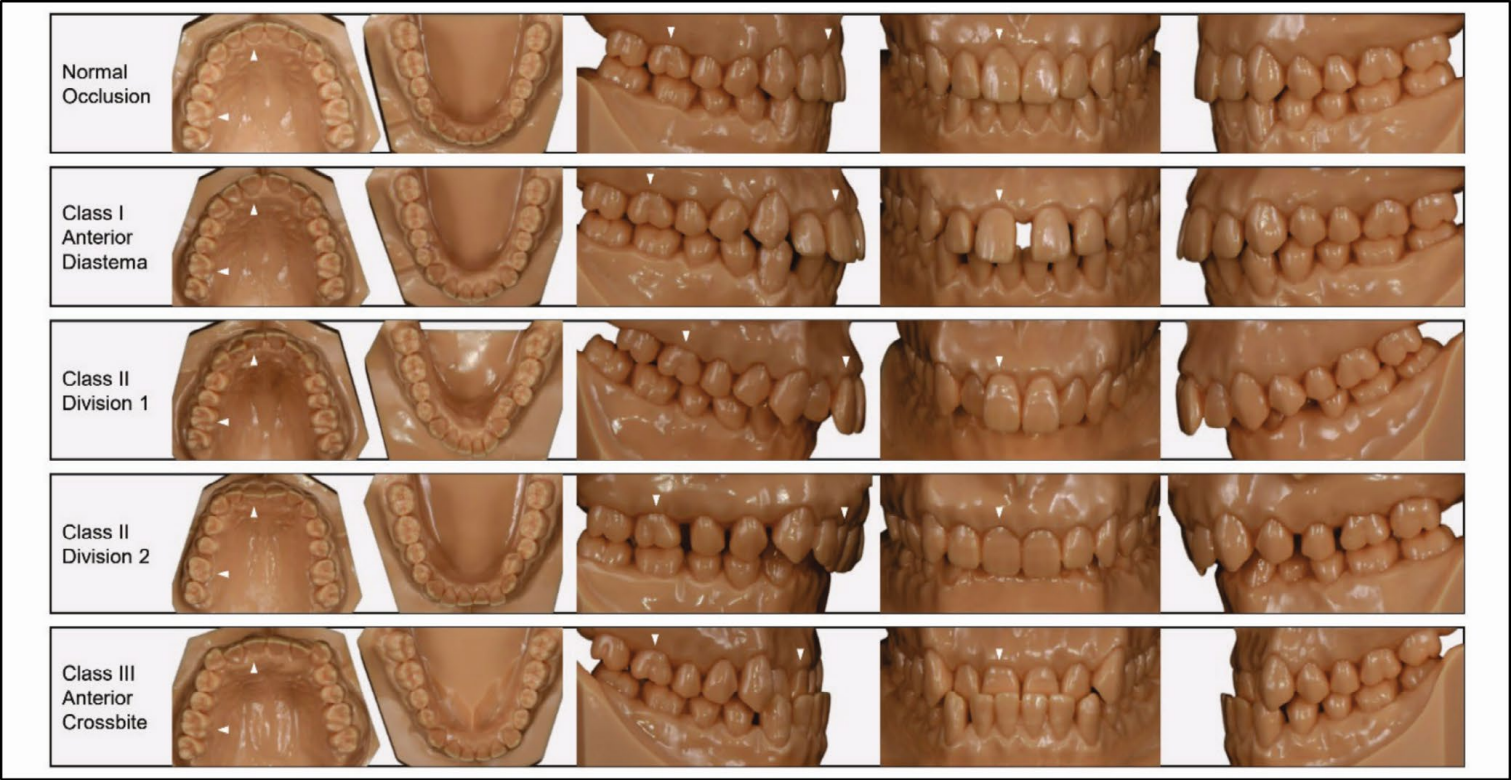
Preoperative views of typodont models representing five different occlusal scenarios. White arrows indicate the maxillary right central incisor and maxillary right first molar.

In this study, five one‐color resin malalignment typodont model sets, based on Angle's classification and produced by a specialized manufacturer of dental educational models (Nissin Dental Products, Kyoto, Japan), were used (Figure 2): a normal Class I occlusion model set (ORT‐1006‐UL‐HD‐28), a Class I anterior diastema model set (ORT‐1008‐UL‐HD‐28), a Class II division 1 deep overbite model set (ORT‐1010‐UL‐HD‐28), a Class II division 2 deep overbite model set with diastema among maxillary posterior teeth (ORT‐1012‐UL‐HD‐28), and a Class III anterior crossbite occlusion model set (ORT‐1014‐UL‐HD‐28). Each model set included a maxillary and mandibular arch. These occlusion types were selected to exemplify a broad spectrum of occlusal relationships frequently encountered in clinical practice.

### 2.3 Reference Tooth Morphology Acquisition

The original occlusion and morphology of each typodont model set were digitized using a desktop scanner (Medit T710, v.2.6.3; Medit, Winnipeg, Canada) with an accuracy of 4 μm (ISO 12836) [31]. The scans were exported in Standard Tessellation Language (STL) format. The tooth morphology of the typodont models, developed by a specialized dental education model manufacturer (Nissin Dental Products Inc., Kyoto, Japan), served as reference data for evaluating the surface deviations of the crown designs.

### 2.4 Crown Preparation for the Maxillary Right Central Incisor and First Molar

For each typodont model set, crown preparations for full‐coverage restorations were performed on the maxillary right central incisor and maxillary right first molar using rotary instruments. A putty index was used to verify the amount of tooth reduction, and all preparations were performed by a single experienced prosthodontist (S.J.K.). For the maxillary right central incisor, the labial axial wall was reduced by 1.5 mm, the palatal axial wall by at least 1.0 mm, the incisal edge by 2.0 mm, and an equigingival chamfer margin was established. For the maxillary right first molar, the preparation guidelines included a minimum axial reduction of 1.0 mm, an occlusal reduction of 2.0 mm, and an equigingival chamfer margin. The amount of tooth reduction was determined to prevent insufficient reduction, which could restrict the CAD‐generated crown designs [32].

### 2.5 Scanning With an Intraoral Scanner

After the crown preparations, the five typodont model sets were scanned using an intraoral scanner (Medit i700, version 3.1.4; Medit, Seoul, South Korea) to capture the prepared abutment tooth structures. Prior to each scanning session, the intraoral scanner was calibrated according to the manufacturer's guidelines to ensure consistent data quality [33]. During intraoral scanning, antireflective scan spray (IP Scan‐spray; IP Division, Haimhausen, Germany) was also applied to the models. In accordance with the manufacturer's recommendations, the scanning process began with the occlusal surfaces and then proceeded to the lingual and buccal aspects. Both the maxillary and mandibular arches, as well as the interocclusal relationship, were captured. Each occlusion‐type model set was scanned 10 times by a single experienced investigator (N.H.M.M.H.), resulting in a total of 10 scan sets per occlusion group (*n* = 10).

### 2.6 Crown Design Using Automated CAD Software Programs

The scan sets obtained by the intraoral scanner were imported into two different automated CAD software programs for crown design. The first software package, a DL‐based AI CAD platform (DL group; Dentbird Crown v. 3.2.1; Imagoworks, Seoul, South Korea), is a web‐based software package using GANs. This software package automates crown design entirely without operator intervention, relying solely on its AI algorithms to generate optimal crown morphology based on the provided scans. Preset parameters were applied across all designs to maintain consistency. The margin line offset was set to 80 μm with an angulation of 45°. To ensure contact on every working cusp and in the central groove area, the occlusal distance was adjusted to 0 μm at each contact area. Proximal contacts were positioned in the occlusal third and extended bucco‐palatally with a 20 μm space. Lastly, the cement gap was set to 30 μm on the internal surface, with an additional 30 μm space. The second software package, a conventional automated desktop‐based software package (CA group; 3Shape Dental System v. 21.2.2; 3Shape, Copenhagen, Denmark), employed proprietary algorithms. For the CA group, the “Auto Workflow” feature of the Dental System (v. 21.2.2; 3Shape, Copenhagen, Denmark) was used, applying the same parameter settings as those used in the DL group. Similarly, in this automated workflow, the operator only selected the abutment tooth, and all subsequent steps, including margin determination and crown design, were performed automatically by the CAD software.

Each automated CAD software program generated one crown per IOS scan dataset (*n* = 10 per group). Crown designs were completed through an automated process without further operator intervention. All auto‐generated crown designs were exported and saved as STL files, each labeled according to the occlusion type and software group.

### 2.7 Evaluation of Crown Designs

#### 2.7.1 Analysis of Surface Deviation From Original Tooth Morphology

Prior to the performance of the three‐dimensional (3D) surface deviation analysis, the STL files of the crown designs were combined with their corresponding dental arches. For the DL group, the STL files of the auto‐generated crown designs and the prepared models were combined using the “Combine” tool in Autodesk Meshmixer software (Autodesk, CA, USA), and the resulting combined STL files were exported. For the CA group, combined STL files of crown designs and prepared models were directly exported from the desktop‐based CAD software.

Surface deviations between the auto‐generated crown designs and the original preoperative tooth morphology (scanned using a desktop scanner prior to tooth preparation) were evaluated using 3D metrology software (Geomagic Control X, v. 2023.1.0, 3D Systems, Rock Hill, SC, USA). STL files of the models and crown designs were imported into the software and initially aligned with the original reference scans using the “Initial Alignment” method. A “Best Fit Alignment” was then conducted on adjacent geometric features: specifically, the left lateral incisor, left central incisor, and right lateral incisor for the maxillary central incisor region; and the right first premolar, second premolar, and second molar for the maxillary first molar region. The areas of interest (auto‐generated crown designs for the central incisor and first molar) were isolated using the “Split” tool. RMS values were calculated to quantify surface deviations between these areas of interest and the corresponding reference tooth morphology of the dentiform. RMS values, representing the average deviation, served as the primary metric for assessing the accuracy of crown designs across different occlusion types.

#### 2.7.2 Clinical Quality Evaluation—FDI Criteria Assessment

The clinical quality of the crown designs was evaluated based on the World Dental Federation (FDI) criteria for indirect dental restorations, an internationally recognized and validated scoring system [34]. The following criteria pertinent to crown design were specifically assessed: functional properties (marginal fit, proximal contact, and anatomical form and contour) and aesthetic properties (surface luster and texture). Each parameter was rated using a five‐point scale: (A) clinically excellent (restoration fulfills all quality standards without any deficiencies); (B) clinically good (restoration is highly acceptable despite minor deviations from optimal criteria); (C) clinically satisfactory (restoration is acceptable with slight imperfections); (D), clinically unsatisfactory (restoration is suboptimal but repairable); and (E) clinically poor (restoration is severely compromised and requires replacement).

The digital crown designs along with their corresponding maxillary and mandibular arches were imported into a 3D object viewer software program (MeshLab 64 bit v2022.02; Visual Computing Lab). Two experienced dentists independently performed the visual inspection of all crown designs by rotating and observing the restorations from multiple perspectives within the software. Prior to the final assessment, calibration between the two examiners was performed. Both examiners were blinded to the software used to generate the crown designs. Any discrepancies or disagreements between examiners were resolved through discussion until consensus was reached [35, 36]. If consensus could not be achieved, a third examiner made the final decision [36].

### 2.8 Statistical Analysis

Statistical analyses were conducted to evaluate the effects of software type (DL and CA groups) and occlusion type (normal occlusion, Class I anterior diastema, Class II division 1, Class II division 2, and Class III anterior crossbite) on RMS values. Prior to the main analysis, the assumptions of normality and homogeneity of variances were tested using the Shapiro–Wilk test and Levene's test, respectively. Both assumptions were violated (*p* < 0.05); thus, a non‐parametric Scheirer–Ray–Hare test was applied to examine the main and interaction effects. If the findings were significant, Dunn's post hoc tests with Bonferroni correction were used to perform pairwise comparisons among the subgroups. For the evaluation using FDI criteria, the distribution of scores among different software (DL and CA groups) and occlusion type groups was analyzed using Fisher's exact test, considering the small sample size and expected frequencies of less than five in multiple cells. Bonferroni correction was applied for multiple comparisons. Statistical analyses of RMS values were performed using R software (version 4.5.0; R Foundation for Statistical Computing, Vienna, Austria), while analyses of FDI criteria scores were conducted using SPSS Statistics software (version 29.0; IBM, Armonk, NY, USA). The significance level was set at *p* < 0.05 for all statistical tests.

## 3 Results

**FIGURE 3:**
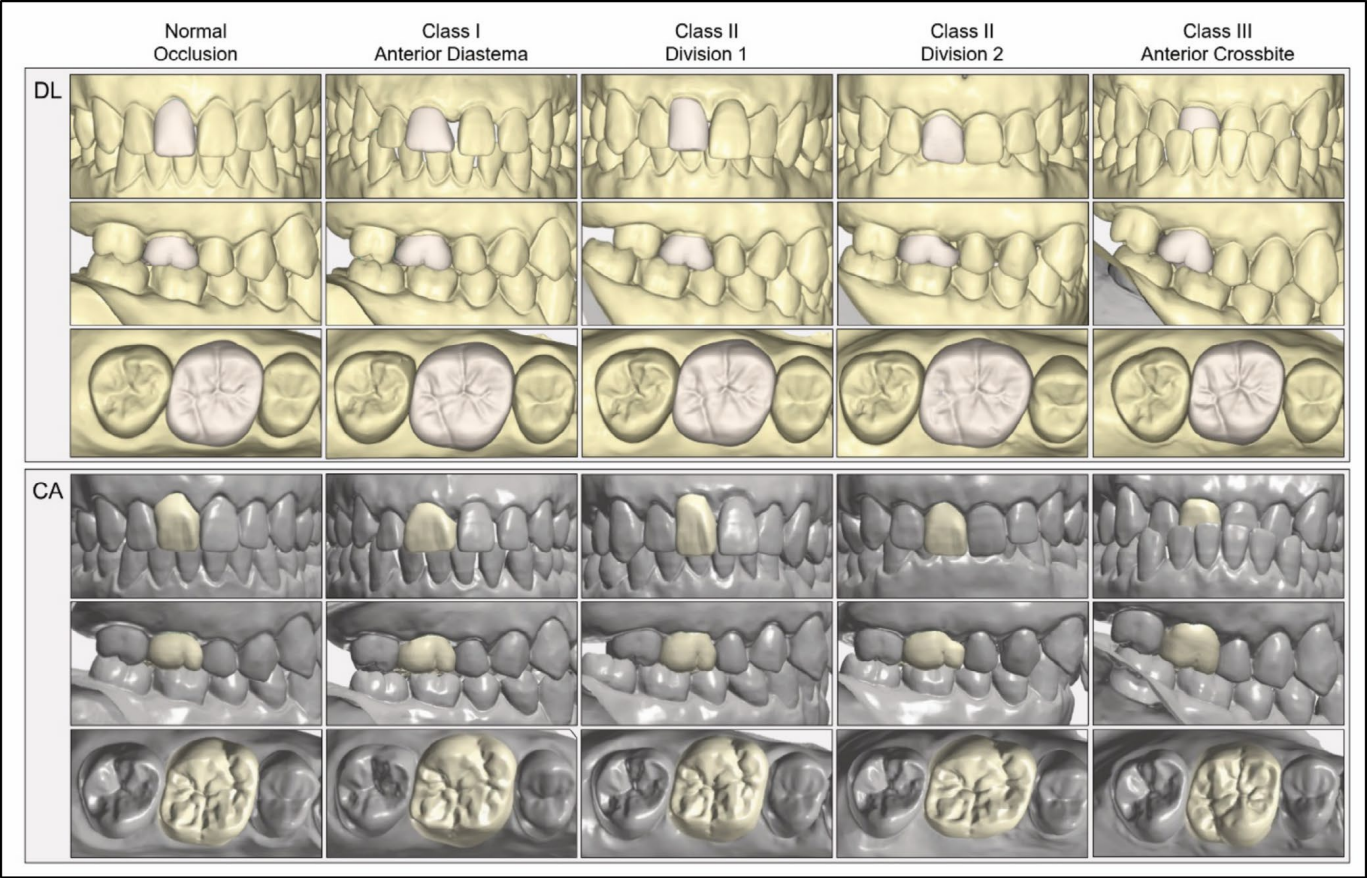
Representative crown designs for the maxillary right central incisors and maxillary right first molars using two automated computer‐aided design software programs. CA, conventional automated computer‐aided design; DL, deep learning‐based computer‐aided design.

Representative images generated through automated design for each occlusal group and software type are shown in Figure 3.

### 3.1 Surface Deviation Outcomes

**FIGURE 4:**
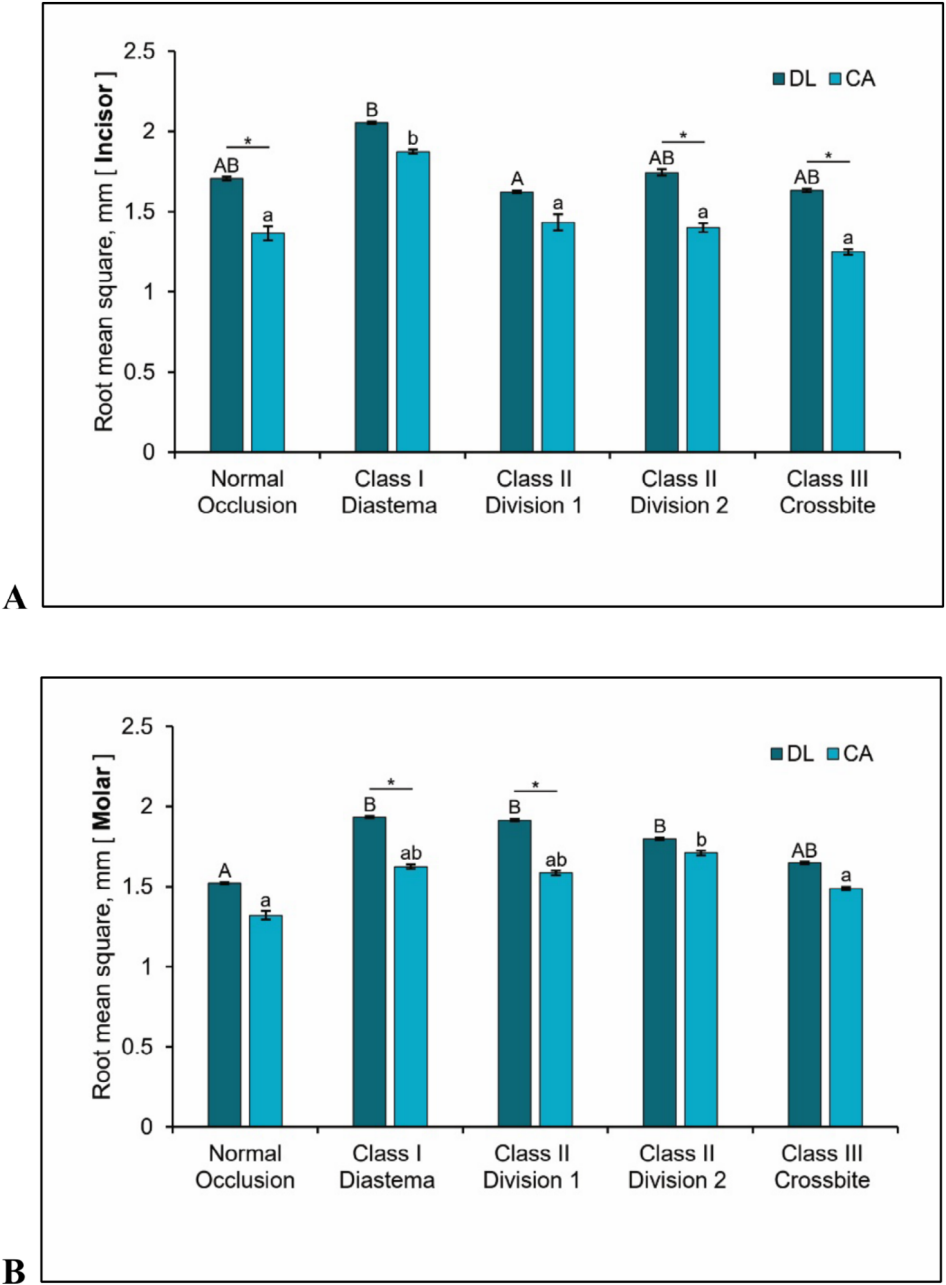
Surface deviations (mm) of the auto‐generated crown designs for the five occlusal scenarios. The same letters signify no significant differences (adjusted *p* > 0.05). Uppercase for DL group, lowercase for CA group. *Adjusted *p* < 0.05. CA, conventional automated computer‐aided design; DL, deep learning‐based computer‐aided design. (A) Maxillary right central incisor. (B) Maxillary right first molar.

In the crown design of the maxillary central incisor (Figure 4A), the main effects of software (*H* = 34.337, *p* < 0.001) and occlusion type (*H* = 53.127, *p* < 0.001) were statistically significant. The interaction effect between software and occlusion was not statistically significant (*H* = 8.538, *p* = 0.074). The mean RMS value for the DL group was 1.752 ± 0.160 mm, while the CA group exhibited a lower mean RMS of 1.464 ± 0.219 mm. Class I anterior diastema showed the highest RMS (1.965 ± 0.093 mm), followed by Class II division 2 (1.573 ± 0.178 mm), Class II division 1 (1.528 ± 0.103 mm), and normal occlusion (1.536 ± 0.177 mm). Class III anterior crossbite exhibited the lowest RMS (1.441 ± 0.197 mm). Dunn's post hoc test for occlusion revealed that Class I anterior diastema differed significantly from all other occlusion types (all *p* < 0.001), while no statistically significant differences were observed among the remaining occlusion types (all *p* > 0.05).

In the crown design of the maxillary first molar (Figure 4B), the Scheirer–Ray–Hare test also demonstrated significant main effects for software (*H* = 34.014, *p* < 0.001) and occlusion type (*H* = 56.058, *p* < 0.001), with no significant interaction effect (*H* = 7.741, *p* = 0.102). The mean RMS value for the DL group was 1.763 ± 0.160 mm, whereas for the CA group, it was 1.546 ± 0.136 mm. Class I anterior diastema again showed the highest RMS (1.780 ± 0.159 mm), followed by Class II division 2 (1.755 ± 0.047 mm), Class II division 1 (1.750 ± 0.170 mm), Class III anterior crossbite (1.568 ± 0.083 mm), and normal occlusion (1.421 ± 0.104 mm). According to Dunn's post hoc test, normal occlusion, which showed the lowest deviation, differed significantly from all other occlusion types (all *p* < 0.001) except for Class III anterior crossbite (*p* = 0.31). Class III anterior crossbite differed significantly from Class I anterior diastema (*p* < 0.01) and Class II division 2 (*p* < 0.01). Other pairwise comparisons among occlusion types did not reach statistical significance (all *p* > 0.05).

### 3.2 Clinical Quality Outcomes (FDI Score)

Table 1 presents the results of crown designs using the FDI criteria for the maxillary right central incisor. DL group crowns had significantly better marginal adaptation results than CA group crowns across all occlusion groups (all *p* < 0.01). The DL group had significantly superior proximal contact point scores to the CA group in the normal occlusion (*p* = 0.003) and Class II division 2 (*p* = 0.011) groups, while no significant differences were observed in other occlusion groups (*p* > 0.05). The proximal contact points criterion was not evaluated in the Class I anterior diastema group due to the inherent presence of diastema between the central incisors. For form and contour, the DL group performed significantly better than the CA group only in normal occlusion (*p* = 0.015). Conversely, the CA group demonstrated better results than the DL group in the Class II division 1 (*p* = 0.033) and Class III anterior crossbite (*p* = 0.033) groups. Most evaluation criteria yielded better scores in the normal occlusion group than in the other occlusion types, particularly for form and contour (*p* < 0.001), where this trend was consistent in the DL and CA groups. The aesthetic properties domain, specifically surface luster and texture, revealed no significant differences between the DL and CA groups across any of the occlusion groups. Both software programs consistently showed clinically acceptable outcomes, receiving scores of A, B, or C for surface luster and texture.

**TABLE 1:** Distribution and comparison of World Dental Federation (FDI) scores for maxillary right central incisor crowns designed using deep learning‐based (DL) and conventional automated (CA) computer‐aided design (CAD) software across different occlusion types.

Domain	Category	Occlusion type	Score	DL		CA		*p*
				*n*	%	*n*	%	
Functional properties	Marginal adaptation	Normal occlusion	A	0	0.0	0	0.0	< 0.001
			B	10	100.0	0	0.0	
			C	0	0.0	1	10.0	
			D	0	0.0	6	60.0	
			E	0	0.0	3	30.0	
		Class I anterior diastema	A	10	100.0	0	0.0	< 0.001
			B	0	0.0	0	0.0	
			C	0	0.0	0	0.0	
			D	0	0.0	2	20.0	
			E	0	0.0	8	80.0	
		Class II division 1	A	10	100.0	0	0.0	< 0.001
			B	0	0.0	0	0.0	
			C	0	0.0	0	0.0	
			D	0	0.0	10	100.0	
		Class II division 2	A	0	0.0	0	0.0	< 0.001
			B	10	100.0	0	0.0	
			C	0	0.0	0	0.0	
			D	0	0.0	7	70.0	
			E	0	0.0	3	30.0	
		Class III anterior crossbite	A	0	0.0	0	0.0	0.007
			B	6	60.0	0	0.0	
			C	1	10.0	0	0.0	
			D	2	20.0	7	70.0	
			E	1	10.0	3	30.0	
		*p*		< 0.001		0.004		
	Proximal contact points	Normal occlusion	A	0	0.0	0	0.0	0.003
			B	7	70.0	0	0.0	
			C	3	30.0	10	100.0	
		Class I anterior diastema	.	Not applicable				
		Class II division 1	A	0	0.0	0	0.0	0.303
			B	1	10.0	4	40.0	
			C	9	90.0	6	60.0	
		Class II division 2	A	0	0.0	0	0.0	0.011
			B	6	60.0	0	0.0	
			C	4	40.0	10	100.0	
	
		Class III anterior crossbite	A	0	0.0	0	0.0	0.211
			B	0	0.0	2	20.0	
			C	10	100.0	7	70.0	
			D	0	0.0	1	10.0	
	*p*		< 0.001		0.02		
	Form and contour	Normal occlusion	A	0	0.0	0	0.0	0.015
			B	4	40.0	0	0.0	
			C	3	30.0	1	10.0	
			D	2	20.0	2	20.0	
			E	1	10.0	7	70.0	
		Class I anterior diastema	A	0	0.0	0	0.0	.
			B	0	0.0	0	0.0	
			C	0	0.0	0	0.0	
			D	0	0.0	0	0.0	
			E	10	100.0	10	100.0	
		Class II division 1	A	0	0.0	0	0.0	0.033
			B	0	0.0	0	0.0	
			C	0	0.0	0	0.0	
			D	0	0.0	5	50.0	
			E	10	100.0	5	50.0	
		Class II division 2	A	0	0.0	0	0.0	1.000
			B	0	0.0	0	0.0	
			C	2	20.0	2	20.0	
			D	7	70.0	7	70.0	
			E	1	10.0	1	10.0	
		Class III anterior crossbite	A	0	0.0	0	0.0	0.033
			B	0	0.0	5	50.0	
			C	10	100.0	5	50.0	
*p*			< 0.001		< 0.001		
Aesthetic properties	Surface luster and texture	Normal occlusion	A	0	0.0	0	0.0	.
			B	10	100.0	10	100.0	
		Class I anterior diastema	A	0	0.0	0	0.0	.
			B	2	20.0	2	20.0	
			C	8	80.0	8	80.0	
		Class II division 1	A	0	0.0	0	0.0	.
			B	10	100.0	10	100.0	
	
		Class II division 2	A	0	0.0	0	0.0	.
			B	10	100.0	10	100.0	
		Class III anterior crossbite	A	0	0.0	0	0.0	.
			B	10	100.0	10	100.0	
*p*		< 0.001		< 0.001		

*Note*: FDI scores indicate clinical quality: A, clinically excellent; B, clinically good; C, clinically satisfactory; D, clinically unsatisfactory; E, clinically poor. *p*‐Values from Fisher's exact test.

Table 2 shows the quality scores of crown designs generated in the DL and CA groups for the maxillary right first molar. Within the functional properties' domain, DL group crowns exhibited significantly better scores than CA group crowns across all criteria and occlusion groups, except for the proximal contact points criterion under Class I anterior diastema, where no difference was observed. DL group crowns consistently received higher ratings, mainly categorized as “clinically good” (score B), across all occlusion types. Conversely, CA group crowns frequently received lower ratings (scores D and E), especially in marginal adaptation. Notably, CA groups frequently generated crown designs rotated by 180°, resulting in the buccal surfaces being positioned lingually and lingual surfaces positioned buccally. This was observed in all occlusion groups except Class III anterior crossbite. This design flaw led to the classification of CA group crowns as “clinically unsatisfactory” (score D) for the form and contour criterion across these groups. Rotated crown designs were not observed in any DL group crowns. Similar to the results of the maxillary central incisor, no significant differences were observed between the DL and CA groups or across occlusion types regarding surface luster and texture within the aesthetic properties domain. Particularly for first molar crown designs, both software programs consistently demonstrated clinically acceptable results (scores A–C) for surface luster and texture, as well as for proximal contact points, across all occlusion groups.

**TABLE 2:** Distribution and comparison of World Dental Federation (FDI) scores for maxillary right first molar crowns designed using deep learning‐based (DL) and conventional automated (CA) computer‐aided design (CAD) software across different occlusion types.

Domain	Category	Occlusion type	Score	DL		CA		*p*
				*n*	%	*n*	%	
Functional properties	Marginal adaptation	Normal occlusion	A	0	0.0	0	0.0	< 0.001
			B	10	100.0	0	0.0	
			C	0	0.0	2	20.0	
			D	0	0.0	6	60.0	
			E	0	0.0	2	20.0	
		Class I anterior diastema	A	8	80.0	0	0.0	< 0.001
			B	2	20.0	0	0.0	
			C	0	0.0	0	0.0	
			D	0	0.0	4	40.0	
			E	0	0.0	6	60.0	
		Class II division 1	A	6	60.0	0	0.0	< 0.001
			B	4	40.0	0	0.0	
			C	0	0.0	0	0.0	
			D	0	0.0	10	100.0	
		Class II division 2	A	10	100.0	0	0.0	< 0.001
			B	0	0.0	0	0.0	
			C	0	0.0	3	30.0	
			D	0	0.0	7	70.0	
		Class III anterior crossbite	A	0	0.0	0	0.0	< 0.001
			B	10	100.0	0	0.0	
			C	0	0.0	0	0.0	
			D	0	0.0	8	80.0	
			E	0	0.0	2	20.0	
		*p*		< 0.001		0.002		

	Proximal contact points	Normal occlusion	A	0	0.0	0	0.0	0.020
			B	9	90.0	3	30.0	
			C	1	10.0	7	70.0	
		Class I anterior diastema	A	0	0.0	0	0.0	.
			B	10	100.0	10	100.0	
		Class II division 1	A	0	0.0	0	0.0	< 0.001
			B	10	100.0	0	0.0	
			C	0	0.0	10	100.0	
		Class II division 2	A	0	0.0	0	0.0	0.020
			B	9	90.0	3	30.0	
			C	1	10.0	7	70.0	
		Class III anterior crossbite	A	0	0.0	0	0.0	< 0.001
			B	10	100.0	0	0.0	
			C	0	0.0	10	100.0	
		*p*		1.000		< 0.001		
	Form and contour	Normal occlusion	A	0	0.0	0	0.0	< 0.001
			B	10	100.0	0	0.0	
			C	0	0.0	0	0.0	
			D	0	0.0	10	100.0	
		Class I anterior diastema	A	0	0.0	0	0.0	< 0.001
			B	10	100.0	0	0.0	
			C	0	0.0	0	0.0	
			D	0	0.0	10	100.0	
		Class II division 1	A	0	0.0	0	0.0	< 0.001
			B	10	100.0	0	0.0	
			C	0	0.0	0	0.0	
			D	0	0.0	10	100.0	
		Class II division 2	A	0	0.0	0	0.0	< 0.001
			B	10	100.0	0	0.0	
			C	0	0.0	0	0.0	
			D	0	0.0	10	100.0	
		Class III anterior crossbite	A	0	0.0	0	0.0	< 0.001
			B	10	100.0	0	0.0	
			C	0	0.0	10	100.0	
*p*			.		< 0.001		
Aesthetic properties	Surface luster and texture	Normal occlusion	A	0	0.0	0	0.0	.
			B	10	100.0	10	100.0	
		Class I anterior diastema	A	0	0.0	0	0.0	.
			B	10	100.0	10	100.0	
		Class II division 1	A	0	0.0	0	0.0	.
			B	10	100.0	10	100.0	
		Class II division 2	A	0	0.0	0	0.0	.
			B	10	100.0	10	100.0	
		Class III anterior crossbite	A	0	0.0	0	0.0	.
			B	10	100.0	10	100.0	
		*p*		.		.		

*Note*: FDI scores indicate clinical quality: A, clinically excellent; B, clinically good; C, clinically satisfactory; D, clinically unsatisfactory; E, clinically poor. *p*‐Values from Fisher's exact test.

## 4 Discussion

The present study identified significant differences in surface deviation (RMS values) and clinical quality outcomes (FDI scores) across different occlusion types and between the two software programs. Notably, crowns in the DL group showed greater geometric deviations from original tooth morphology but achieved superior clinical ratings to crowns in the CA group. Furthermore, design outcomes differed significantly among the various occlusal scenarios, with normal Class I occlusion generally yielding the most favorable results. Malocclusions, such as Class I anterior diastema and Class II occlusion, presented more substantial challenges, leading to greater design discrepancies and reduced clinical quality scores. Consequently, both null hypotheses, that no differences would exist among the five occlusion types and that the two automated CAD software programs would perform equally, were rejected.

For the anterior tooth, both the DL and CA groups showed the highest RMS values in Class I anterior diastema. In agreement with this, both the DL and CA groups had the worst FDI scores for form and contour in Class I anterior diastema. Regardless of the software, auto‐generated crown designs for the incisor in Class I anterior diastema exhibited higher surface discrepancies than the other occlusion types. The design images from both software types confirmed that crown designs appeared wider than the symmetrical tooth. This suggests that both software programs automatically filled the diastema space in the original model during the design process, leading to greater deviations from the original tooth form and poor aesthetics. This result highlights the necessity for further refinement of automated CAD software designs in patients with diastema to improve crown design outcomes. Çakmak et al. compared the design outcomes (crown morphology, function, and aesthetics) of auto‐generated anterior crowns created using the same DL‐based CAD software program (DL group) with those fabricated by a technician using conventional dental CAD software without automated support, in a normal occlusal scenario (no deep overjet or overbite) [20]. Their results suggested that auto‐generated anterior crowns demonstrated promising outcomes with clinically acceptable morphology and aesthetics [20]. Because in the present study both software packages applied automated design without a technician‐based control group, direct comparison with this previous study is difficult.

For the posterior tooth designs, similar to the anterior tooth results, both DL and CA groups exhibited higher RMS values in the Class II division 2 group, in which a diastema existed adjacent to the restored tooth, compared to the normal occlusion group. However, in contrast to the anterior tooth results, the DL group showed clinically acceptable FDI scores in Class II division 2. This is likely because the CAD software created contact points by automatically filling the preoperative diastema that was present between the first molar and second premolar (Figure 2) during crown design (Figure 3). In the anterior teeth, the software's mechanism of creating contact areas in the diastema region resulted in asymmetrically sized central incisors, leading to clinical unacceptability due to aesthetic concerns. Conversely, in the posterior region, which is less aesthetic‐sensitive, the filling of diastema spaces to create contact points can yield clinically superior outcomes by preventing food impaction and tooth drifting [37]. Thus, the posterior teeth in the Class II division 2 group demonstrated favorable FDI scores. This observation underscores an essential consideration in the development of AI‐based CAD software: the same design mechanism can produce contradictory outcomes depending on tooth position (anterior versus posterior) or specific occlusal scenarios. Future software development should incorporate these nuances to optimize clinical outcomes across diverse clinical conditions.

The DL group demonstrated lower surface deviation values when designing posterior crowns in normal occlusal scenarios than in those designed for malocclusion models, along with clinically acceptable FDI scores (A–C) across all evaluation criteria. Although greater surface deviations were observed in other occlusal scenarios, the DL group still provided clinically acceptable FDI scores in all areas. In contrast, the CA group occasionally generated crowns rotated by 180° (Figure 3), resulting in clinically unacceptable FDI scores, particularly in the form and contour criterion. The variations in performance between these automated CAD software programs may be caused by the different design algorithms used [10, 18, 19]. The web‐based AI software (DL group) used deep learning and CAD algorithms to design the crown, resulting in better outcomes [19]. The results in the present study suggest that recent advancements in DL‐ and GAN‐based artificial intelligence allow current DL‐driven software to reliably produce clinically acceptable posterior crown designs across various occlusal relationships and malocclusion scenarios.

Interestingly, crowns from the DL group demonstrated superior clinical quality despite exhibiting larger morphological deviations, possibly due to slight over‐contouring. A potential explanation is that the AI‐driven design strategy used in the DL group prioritizes critical functional parameters, such as complete marginal seating and adequate contact tightness. Previous research on posterior implant crowns similarly reported that DL‐based designs produced occlusal anatomy and contact profiles comparable with clinician‐designed crowns, albeit with greater overall volume deviations [17]. Another recent study comparing AI‐based crown design software, including a DL group, with conventional CAD reported that AI dramatically improved design efficiency (requiring approximately 25% of the manual design time), yet exhibited lower morphological accuracy than expert technician designs [28]. CA group crowns exhibited smaller morphological deviations yet did not outperform DL‐generated crowns in functional outcomes such as marginal adaptation, form, and contour quality. Thus, perfect geometric matching (low RMS) alone is not the sole determinant of clinical success. Clinically, crowns with excellent seating, adaptation, and occlusal relationships are more likely to be successful in the long term than those merely matching geometric ideals. Furthermore, a recent GAN‐based study achieved remarkably lower morphological discrepancies in crown designs than traditional CAD but emphasized that such geometric accuracy must be balanced against functional fit considerations [9]. The current findings similarly suggest that slight over‐contouring in the DL group's designs, despite increasing RMS error, may enhance clinical adaptation, highlighting a practical trade‐off between morphological precision and clinical functionality, especially for posterior teeth. However, in anterior restorations, form, contour, and aesthetics become critical determinants of clinical success, indicating that further refinement of automated design strategies may be required in these cases.

To the best of the authors' knowledge, no previous comprehensive evaluation of the impact of occlusion types on AI‐generated crown designs has been conducted. However, the in vitro nature of this study may not fully capture the complexities encountered in clinical scenarios, such as patient‐specific variations in the oral environment and occlusal dynamics. Additionally, this study focused exclusively on two automated CAD software programs; thus, the findings might not be generalizable to other software platforms. Furthermore, establishing a standard reference tooth morphology was challenging. This study used typodont models manufactured by a specialized dental educational model manufacturer (Nissin Dental Products, Kyoto, Japan) as references for surface deviation analysis. However, accurately defining an ideal tooth morphology as a reference for surface deviation assessment across diverse malocclusion scenarios remains inherently challenging. To overcome these limitations, this study included both surface deviation assessments and FDI criteria evaluations, allowing clinicians to assess the clinical quality and appropriateness of the designs. Future prospective clinical studies incorporating a wider variety of occlusal relationships, a broader range of tooth types, evaluation of the accuracy of occlusion itself, and validation in real‐world patient settings are needed to address these limitations and confirm the clinical utility of AI‐generated crown designs.

## 5 Conclusion

Within the limitations of this current study, it was concluded that:

1. Crowns designed using automated CAD software differ significantly in both geometric accuracy and clinical quality, particularly in clinically challenging scenarios, such as diastema and other complex malocclusions.
2. Although current DL‐based CAD software generally shows superior clinical outcomes compared to CA software especially for molar designs, further software advancements are necessary to consistently address these limitations and enhance clinical performance.

## Author Contributions

**Nan Hsu Myat Mon Hlaing:** conceptualization, methodology, investigation, software, data curation, formal analysis, visualization, and writing – original draft preparation. **Gülce Çakmak:** conceptualization, methodology, validation, writing – original draft preparation, writing – reviewing and editing. **Duygu Karasan:** conceptualization, methodology, validation, writing – reviewing and editing. **Sung‐Jin Kim:** conceptualization, investigation, data curation, writing – reviewing and editing. **Irena Sailer:** conceptualization, methodology, validation, supervision, writing – reviewing and editing. **Jae‐Hyun Lee:** conceptualization, methodology, resources, visualization, funding acquisition, supervision, project administration, writing – original draft preparation, writing – reviewing and editing.

## Conflicts of Interest

The authors declare no conflicts of interest.

## Data Availability

The data that support the findings of this study are available from the corresponding author upon reasonable request.
